# Mouse Models to Interrogate the Implications of the Differentiation Status in the Ontogeny of Gliomas

**DOI:** 10.18632/oncotarget.319

**Published:** 2011-09-03

**Authors:** Diana Marcela Muñoz, Abhijit Guha

**Affiliations:** ^1^The Arthur and Sonia Labatt Brain Tumor Research Centre, Hospital for Sick Children Research Institute, University of Toronto, Toronto, Ontario, Canada, M5G 1L7; ^2^Division of Neurosurgery, Toronto Western Hospital, University of Toronto, Toronto, Ontario, Canada, M5T 2S8

**Keywords:** Glioblastoma Multiforme (GBM), Tumor Heterogeneity, Tumor Microenvironment, Brain Tumor Stem Cells (BTSC)

## Abstract

Glioblastoma Multiforme (GBM) is the most common and lethal of human primary central nervous system (CNS) tumors, with a median survival of 14-16 months despite optimal surgery, radiation and chemotherapy. A reason for this dismal prognosis is insufficient understanding of the ontogeny of GBMs, which are highly heterogeneous at a pathological level. This pathological diversity, between and within GBMs as well as varying grades of gliomas, has not been fully explained solely on the grounds of oncogenic stimulus. Interaction with the tumor microenvironment is likely a source of this pathological heterogeneity, as well as the inherent characteristics of the tumor cell of origin. Currently, controversy exists on whether the initial transformed cell is a differentiated astrocyte, progenitor or neural stem cell. Putative Cancer Stem Cells (CSCs), which have features of normal stem cell plus the ability to recapitulate the tumor phenotype in vivo in small numbers, have been identified from a variety of solid human cancers, including GBMs. Evidence suggesting that regions harboring normal stem cells in the adult CNS, such as the subventricular zone and the dentate gyrus, are more prone to viral and chemical oncogenesis, is supportive of the hypothesis that brain tumors arise from stem cells. However, it is still to be determined whether the appearance of brain tumor stem cells (BTSC) is the cause or consequence of tumor initiation and progression. This review discusses emerging evidence highlighting the relevance of the state of differentiation and regional heterogeneity in the ontogeny of GBM. This is an area of high interest in cancer in general, with potential significant therapeutic and prognostic implications.

## INTRODUCTION

Gliomas compose almost 60% of total human primary CNS malignancies [1, 2]. These tumors are recognized as a heterogeneous group of neoplasms that differ in location, morphological features, tendency for progression and response to therapy. The malignant Grade IV Glioblastoma Multiforme (GBM) is the most common, with an associated median survival of 14-16 months [2, 3]. Despite progress in research on the molecular aspects of malignant gliomas the prognosis of these brain tumors continues to be dismal. We believe that research on the ontogeny of GBMs, presents an opportunity for improving our understanding and treatment of this disease.

**Figure 1:**
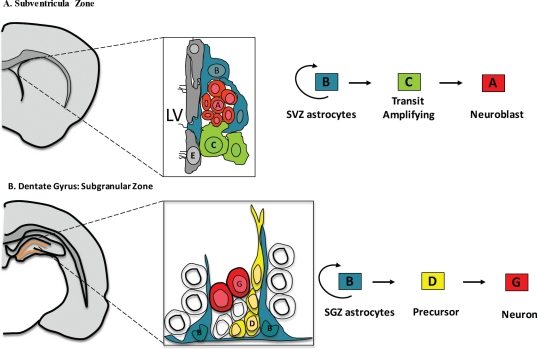
Cellular composition and cytoarchitecture of germinal regions within the adult brain **A**. Frontal schema of the Subventricular zone in the adult mouse brain and the cell types from which it is composed.Multi-ciliated ependymal cells (E, Gray) line the lateral ventricle (LV). SVZ astrocytes (B, Blue) are primary progenitors which generate neuroblast (A, Red) through a rapidly dividing transit amplifying cell (C, Green). Occasionally Type B cells extend a short primary cilium into the ventricle. **B**. Frontal schema of the Subgranular Zone within the dentate gyrus of the adult mouse brain. The cell types that compose the SGZ include, SGZ astrocytes (B, Blue), the in-vivo primary precursors of new granule neurons (G, Red) through an intermediate precursor Type D cell (D, Yellow).

For decades, it was widely assumed that differentiated glia were the only cells capable of transformation as the adult brain was thought to be mitotically inactive [4]. The demonstration of functional neurogenesis in the adult CNS[5-8] provided new possibilities for the candidate cell of origin of CNS neoplasms. These progenitor populations reside in multiple regions of the adult brain, including the subventricular zone (SVZ) and the dentate gyrus within the hippocampus [5, 9-12] (Figure 1). The largest of these germinal regions in humans, the SVZ, has long be proposed as a source of gliomas, as many are either periventricular or contiguous with the subventricular zone [13-16]. Additionally, cells that express the NG2 proteoglycan, distributed throughout the brain have also been proposed as the cells origin of gliomas [17, 18]. In summary, the cell of origin maybe differentiated glial and/or neuro-glilal progenitor stem cells subjected to a variety of transforming molecular alterations.

Conditional mouse modeling combined with lineage tracing techniques makes it possible to target these distinct neurogenic regions in adults, as well as cell populations at different stages of their differentiation. These and other reagents have facilitated experiments towards a better understanding of the ontogeny of GBMs, which have underscored the relationship between gliomagenesis and brain development. A relationship further highlighted by the The Cancer Genome Atlas (TCGA) which sub-classified GBMs to four gene signature profiles (Classical, Neural, Proneural and Mesenchymal), which are characteristic of distinct stages, prognosis and patterns of differentiation [19, 20]. This reinforces the need to view the tumourigenic process of gliomas, in the context of normal brain development.

## DEFINITION OF CNS CELL TYPES AND THEIR REGIONAL DIVERSITY

CNS development proceeds in a hierarchical manner; whereby, mature differentiated cells such as neurons and the varying glial subtypes (astrocytes, oligodendrocytes, ependymocytes) arise from CNS stem cells. These CNS stem cells arise from the pseudostratified neuroepithelium that lines the cerebral ventricles early in embryonic development and stains positive for Nestin [21]. By the onset of neurogenesis, these neuroephitelial cells are replaced by a more differentiated neural stem cell, the radial glia cell [22, 23]. Radial glia has a more glial like pathological profile with expression of BLBP (brain lipid binding protein)[24]. By adulthood, most CNS progenitors have disappeared, however, stem cells capable of giving rice to differentiated CNS cell types are retained in distinct anatomical regions, including the SVZ lining the lateral ventricles[9, 25] and the dentate gyrus within the hippocampus [11].

The subventricular zone (SVZ) is the largest germinal region, and it exhibits a high degree of organization, Figure 1A. It is composed of Type B cells, slowly dividing SVZ astrocytes that function as primary progenitors, giving rise to actively proliferating intermediate progenitors known as Type C cells [26]. Type C cells give rise to immature neuroblasts or Type A cells, which migrate in chains to the olfactory bulb, where they differentiate into distinct interneuronal populations[27]. Type B cells are marked by the expression of GFAP and they retain important properties of radial glia, thus it has been postulated that radial glia transforms directly into Type B cells[23-25].

Similarly, neural stem cells within the hippocampus seem to have a radial glial origin, Figure 1B. The Subgranular Zone (SGZ) within the dentate gyrus of the hippocampus, is composed of two types of dividing cells, astrocytes and darkly stained small cell with small basophilic nucleus [28]. Similar to the SVZ, radial astrocytes function as the primary precursors for new neurons through an intermediate progenitor, referred to as Type D cells [28].

A third population of CNS precursors in the adult brain, is a subpopulation of NG2 expressing cells shown to play a glial precursor role. The NG2 proteoglycan is believed to be an in vivo marker for oligodendrocytes progenitors found in the developing brain [17, 18, 29, 30]. However, a significant proportion of NG2-expressing cells remain in the adult CNS following the end of gliogenesis. These cells continue to proliferate and can give rise to neurons and macroglia, as opposed to the SVZ and SGZ progenitors described above. NG2 expressing cells are found throughout the adult brain [18], however, the heterogeneity of this population has not been adequately addressed in the adult CNS. While NG2 cells continue to divide in the adult CNS it is not clear what function they serve once myelination is complete. Future studies should elucidate the functional importance of NG2 in a variety of cell functions and shed light on the role NG2-expressing cells play in the intact and diseased CNS.

All of the neural stem and progenitor populations described above have different proliferative capacities and varying ability to generate astrocytes, oligodendrocytes and neurons that may reflect distinct patterns of gene expression. Thus regional differences in neuroglial precursor populations may dictate a significant component of CNS tumor gene expression and biology.

## IMPLICATIONS FOR CNS TUMORIGENESIS

All forms of cancer arise from the acquisition of genetic alteration, leading to several aberrant biological properties required for transformation. These biological properties include, sustained proliferation, evading growth suppression, resisting cell death, enabling replicative immortality, inducing angiogenesis and activating invasion and metastasis [31]. In the adult brain, existing normal neural stem cells and progenitor elements represent the path of least resistance to tumorigenesis. These cells already have the genetic machinery for promoting mitosis, to resist apoptosis and senescence [32], making them an attractive candidate for the cell of origin for gliomas. Nevertheless, any cell in the hierarchy with proliferative capacity could serve as a cell of origin in gliomas, if it acquires mutations that re-instigate self-renewal capacity and prevent differentiation to a post-mitotic state. The normal lineage hierarchy in the CNS can serve as a framework for understanding cells of origin in GBM's, by comparing expression signature of normal cell populations with those of different tumor subtypes.

Comparative gene expression analysis in leukemias show that the different forms of leukemias appear to arise from distinct points in the hematopoietic lineage, that are susceptible to specific translocations. Furthermore, the biology behind this translocations-lineage stage mechanism might lie in the capacity of translocations to activate key leukemogenic programs [33, 34], reinforcing the idea that cancers arise from specific combinations of mutations and susceptible cell types. In the CNS this idea has been shown to hold true in ependymomas, which carry transcriptome, and DNA copy alterations that correlate with tumor location [35, 36]. Similarly gene expression profiles of medulloblastomas carve these cancers into clinically and molecular distinct subgroups [37]. Genomic profiling of GBMs has revealed patterns of molecular changes with tumor subclasses that harbor distinct underlying biology and clinical prognosis. One such study used correlative expression profiling to subdivide gliomas into groups based on similarities to known cell types. The resulting groups were termed Pro-neural, Proliferative and Mesenchymal [38]. A more recent analysis of one larger TCGA GBM data set, classified GBMs as Pro-neural, Neural, Classical and Mesenchymal. These subclasses are strongly associated with genomic abnormalities in *PDGFR*, *IDH1/IDH2*, *EGFR* or *NF1* genes respectively [19, 20, 39]. However, the link between precursor cells to genomic classification of GBMs has not been firmly established. First, these analysis were on bulk GBM tumors, which show phenotypic plasticity or differentiation during neoplastic progression, Hence, the molecular signatures of bulk tumor cells may not precisely reflect the true cell of origin in normal tissue. To overcome this, rather than analyzing terminal stage tumors, this kind of studies should be carried out on tumors at different stages of their malignant progression.

There exists debate about the Cancer Stem Cell (CSC) hypothesis and the existence of BTIC in brain tumors. Unlike leukemias discussed above, there is no singular cell surface or genetic signature of the BTIC. Initial enthusiasm with CD133 has recently been questioned by studies demonstrating that alternations in mitochondrial function among glioma cells induce CD133 expression[40]. Conversely, replacement of dysfunctional mitochondrial genes can reverse CD133 expression[40]. These results suggest that CD133 expression in gliomas is triggered as a response to environmental stress, questioning the reliability of CD133 as a BTIC marker. Although the CSC hypothesis seems to be supportive of the hypothesis that brain tumors arise from the transformation of normal neural stem cells, these two concepts are distinct and in most instances the identity of the cell of origin may differ substantially from that of the BTIC.

Another but not mutually exclusive hypothesis is that some GBMs can arise from the transformation of terminally differentiated glia. Retroviral transduction of INK4a/Arf-/- mature astrocytes with a constitutively active mutant EGF receptor (EGFRvIII), prevalent in human GBMs, induces astrocyte de-differentiation and GBM formation[41]. A phenomenon, which is also observed, when GFAP positive cells are infected with a PDGF expressing retrovirus using the RCAS/tva system [42]. In addition, overexpression of the transcriptional factor c-myc, in astrocytes results in the down regulation of the astrocytic marker GFAP and upregulation of Nestinpanicker[43, 44], suggestive of transformation. However one of the most relevant peaces of evidence comes from the demonstration, that adult fibroblast can be reprogrammed to a pluripotent stem cell state by transfection of a small number of transcription factors [45], suggesting that differentiated cells can be endowed stem cell like properties. Arguably, similar process might be relevant during brain tumor pathogenesis. This controversy can only be settled with in vivo studies to asses the propensity of cell populations to act as cell of origin. Mouse models of oncogenesis have been pivotal in this regard, and have been of great insight into the ontogeny of GBMs

## LESSONS FROM MOUSE MODELS

To elucidate the ontogeny of gliomas conditional and somatic strategies coupled to lineage tracing techniques have come to the forefront, as summarized on Table 1. Conditional strategies were developed to allow for the control of gene expression in both a tissue and or time specific manner. For example, Tet/Tamoxifen-regulatable and Cre inducible genes, can allow for the control of the timing, duration and tissue compartment of gene expression or inactivation. In addition, several methods for somatic gene transfer have been developed using retroviral or adenoviral delivery of Cre recombinase. This approach, allows for a more accurate modeling of human cancers as most mutations are thought to occur somatically in a single cell or small group of specific cell types. All of the above mentioned strategies have been used to study the effects of different GBM relevant mutations and their cooperation at different developmental time-points. However, the most convincing data will come from experiments that clearly demonstrate the ability of a cell to get transformed and initiate tumor development. Towards this end, linage-tracing techniques are used to label and follow cells of known type in the CNS for changes. As reviewed below the use of these powerful techniques has shed some light into the cellular origins of GBMs.

**Table 1:** Candidate cell of origin identified in GBMs by targeting distinct cellular compartments and or anatomical regions

Mouse model	Genetic model	Promoter-Cre construct	Anatomical region	Cell of origin
**Germline**				
	^V12^H-Ras activation	hGFAP-Cre	NA	SVZ Multipotent progenitor[47]
	p53, Nf1 inactivation	hGFAP-Cre	NA	SVZ Multipotent progenitor[49]
	p53,Pten,Nf1 inactivation	Nestin-CreERT2	NA	Multipotent progenitor[50]
	Pten, Rb1, p53 inactivation	GFAP-Cre ERT2	NA	Multipotent progenitor and Astrocytes[53]
	p53,Nf1 inactivation	GFAP-Cre	NA	Oligodendrocyte precursor[53]
	p53,Nf1 inactivation	NG2-Cre	NA	Oligodendrocyte precursor[53]
	Mutant p53	GFAP-Cre	NA	Neural progenitor, Transit-amplifying cell[66]
**Somatic**				
	Nf1, p53, Pten inactivation	Adeno-Cre	SVZ, Striatum, Cortex	Multipotent progenitor[50]
	Rb, p53, Pten inactivation	Adeno-Cre	SVZ, Striatum	Multipotent progenitor, transit-amplifying cell[60]
	Kras, AKT activation	GFAP-tva	NA	Multipotent progenitor[62]
	Kras, AKT activation	Nestin-tva	NA	Multipotent progenitor[62]
	^V12^Hras, AKT activation, p53 inactivation	GFAP-Cre	SVZ, Cortex, Hippocampus	Multipotent progenitor[63]
	Ink4a/Arf, Pten, RCAS-PDGF	RCAS-Cre/ GFAP-tva	SVZ, striatum, cerebellum	Multipotent progenitors and astrocytes[65]
	Ink4a/Arf, Pten, RCAS-PDGF	RCAS-Cre/Nestin-tva	SVZ, striatum, cerebellum	Multipotent progenitors and astrocytes[65]
**Primary cultures**				
	P16^Ink4a^/P19^ARF^Bmi1 inactivation, mutant EGFR	NA	Cortex, SVZ	Neural progenitor and astrocytes[67]

### Germline strategies: Targeting defined cell populations at specific developmental time-points

The first germline derived mouse models for brain tumors, were models that over expressed viral oncogenes [46]. However it was soon realized that most are embryonically lethal. Overtime, genetic models evolved to the use of conditional strategies and genes where driven by cell type specific promoters [47, 48]. Using these strategies, brain tumor models have been developed by altering signaling pathways that are disrupted in human gliomas including Rb, Ras, AKT, Pten, NF1 amongst others.

To study the role of Ras signaling in gliomas, a mouse model carrying a constitutively active Ras (V^12^Ras) under the control of a human hGFAP promoter was developed [49] by our group. This model highlighted the importance of Ras gene dosage during tumor development, as high expression levels lead to the development of multifocal GBMs within two weeks after birth. As opposed to moderate levels of expression, which lead to 50% of the mice developing high-grade astrocytomas by three months of age [49, 50]. This model more accurately represents secondary GBMs, as these mice are born normal but develop low-grade astrocytomas by 3-8 weeks of age [49]. Upon micro-dissection, the low-grade gliomas harbored p53 mutations, similar to low grade astrocytomas in humans. Currently we are deciphering if they also harbor mutations in IDH, which are highly prevalent in human low-grade astrocytomas. At 12 weeks highly invasive GFAP-/Nestin + high-grade astrocytomas develop, a progression that is accompanied by additional changes including overexpression of EGR, MDMT, loss of Pten and p16 and p19 [50], all of which are known genetic markers associated with malignant human gliomas. The cell of origin in this model is uncertain, but likely from GFAP positive progenitors in the SVZ (unpublished data).

In a different model, Zhu et al. used the same hGFAP promoter to undertake Cre induced deletion of *p53* and *Nf1*, showing that early inactivation of *p53* gene cooperates with *Nf1* loss to induce GBM development [51]. Similar to the Ras model, the earliest identifiable area of a tumor was confined to the SVZ. These two models suggest that either a specific cell type or the microenvironment in this region provide a favorable niche for the growth of early tumor cells [51].

However, the hGFAP promoter used in both models is expressed in both stem cells and white matter astrocytes. Thus whether the tumors arose from a stem or progenitor population or a differentiated astrocyte cannot be definitely determined. To address this, Alcantarallaguno et al. used a tamoxifen inducible Nestin-Cre to inactivate the tumor suppressor *Pten*, *Nf1* and *Tp53* at different developmental time points. During early embryogenesis, the Nestin promoter produced a broad pattern of expression similar to the hGFAP transgene [52]. However, induction during adulthood leads to recombination almost exclusively in the neural stem cell population of the SVZ and SGZ [52, 53]. In this model, Induction of recombination during early embryogenesis lead to the development of tumors at rates similar to the hGFAP-Cre; *Tp53*; *Nf1* glioma model, with a median survival of 16 weeks. In contrast, tumors observed when recombination was induced at adulthood, had a median survival of 46 weeks [52]. This observation suggests that early progenitors seem to be more susceptible to transformation and at least in this model the cell of origin seems to reside in the neural stem/progenitor populations of the adult brain.

Recently, a tamoxifen inducible GFAP promoter was used to induce the deletion of *Pten*, *Rb1* and *Tp53* in astrocytes in a limited population of neural progenitors in the adult brain [54, 55]. This model showed selective cooperativity among tumor suppressors. *TP53* deletion was required for the formation of high-grade astrocytomas induced by the deletion of *Pten*, *Rb1* or combined *Pten Rb1* deletions [55]. Many tumors were contiguous with regions rich in neural progenitor cells such as the SVZ, the rostral migratory stream and subgranular zone of the dentate gyrus. However, there were a significant 20% of gliomas that developed in sites independent of these proliferative niches. This suggests that astrocytomas induced by this set of mutations can develop outside proliferative niches, albeit at lower efficiency.

Despite all the insights from the models discussed above, the true cell of origin is still controversial. This mainly due to the fact that the cell of origin is difficult to identify by analyzing cells within terminal stage tumors, whose genetic identity could be concealed by the acquired plasticity. A recent study aimed at addressing this issue analyzed proliferative abnormalities in distinct lineages prior to malignancy [56]. Using a mouse model approach that allows simultaneous labeling and gene knockout in clones of somatic cells [57, 58], Liu et traced the growth of individual lineages that descended from a mutant neural stem cell lacking* p53* and *Nf1* expression. After recombination, a dramatic overexpansion and aberrant growth of oligodendrocytes progenitors (OPC), but not of neural stem cells or other lineages was observed. Further transcriptome and marker staining analysis confirmed upon tumor formation the OPC nature of the tumor cells. This demonstrates that in *p53/Nf1* mutant driven gliomas, mutations may initially occur in either NSC or OPC, but only OPC provides the susceptible cellular context needed for transformation [56]. This highlights the concept that cells initially acquiring a mutation might not be the cell of origin, but rather its descendants who acquire additional transforming alterations.

### Somatic strategies: Targeting defined anatomical regions in the CNS

Germinal regions such as the SVZ have long been proposed as more gliomagenic, as this region is more susceptible to viral and chemical oncogenesis [59-61]. In canine and rodent brains for example, avian sarcoma viral transformation [60]or systemic exposure to the carcinogen N-ethyl-N-nitrosourea, leads to tumor formation, preferentially in the proliferative SVZ rather than in non-proliferative regions of the brain [59]. This observation suggested that gliomas could originate from the neural stem cell population that resides in the SVZ; however, the genetic alterations necessary for transformation of these stem/progenitor cells are recently being unraveled.

Work by Alcantarallaguno et al. demonstrates that viral mediated Cre-recombinase targeting of the SVZ in an *Nf1/p53 flox* background induced astrocytoma formation with 100% penetrance [52]. Conversely, targeting regions such as the striatum did not lead to tumor formation. Targeting of the SVZ was carried out at two different developmental time-points, early postnatal and adulthood. Tumor suppressor inactivation in the SVZ of both early postnatal and adult stages induced astrocytoma formation, however, the early postnatal injected mice developed more and extensive gliomas compared to adult mice[52].

Using mice bearing conditional alleles of *Rb, p53 and Pten* Jacks et al showed that the initial combination of genetic mutations in the SVZ dictates the pathogenesis and phenotype of the tumors that develop. In these mice, recombination of *Pten/p53* gave rise to gliomas, whereas deletion of *Rb/p53* generated primitive neuroectodermal tumors (PNET) [62]. This model raises questions regarding the relationship between state of differentiation and the oncogenic growth signal. Is it that different populations of stem/progenitor cells are transformed by a specific set of mutations? Or is it that the different tumor types developed from the same “Tumor progenitor” but weather a glial or primitive neuroectodermal differentiation is observed is dependent on the genetic alteration. Further optimization of this techniques using Cre reporter mouse lines in combination with promoter specific Cre drivers will allow us to look at this questions.

Other retroviral-based approaches such as the RCAS system have allowed lineage tracing of specific cell populations through the process of tumorigenesis [63]. The virus infects mammalian cells that express the subgroup a receptor for the RCAS virus (tv-a), which is not encoded by the mammalian genome. This system has been used to transfer constitutively active mutant forms of Kras and AKT to both Nestin positive progenitor cells and GFAP positive astrocytes [64]. Although neither activated Kras nor AKT alone is sufficient to induce GBM formation, the combination is sufficient to induce high grade gliomas with histological features of human GBMs. Furthermore, the gliomas were found in the Nestin drive tv-a neural progenitors, but not in the differentiated GFAP-tv-a astrocytes [64], highlighting the importance of both cooperating mutations as well as the differentiation status of the cell of origin. Similar results were observed when a Cre loxP controlled Lentiviral vector was injected into specific anatomical regions to induce expression of activated H-Ras and AKT in GFAP+ astrocytes in the adult mice [65, 66]. In this model, high-grade gliomas developed only when the SVZ or hippocampus were targeted. Tumors were rarely detected when less proliferative areas such as the cortex were targeted [65].

In contrast to targeting the neural-glial progenitor population, a study used the RCAS system, with injection of the RCAS-PDGF + RCAS-Cre virus into various regions of a Nestin-tva; Ink4aARF null; Pten flox adult mice. These regions included the SVZ, left and right cerebral hemispheres and cerebellum, with development of gliomas in all of the areas targeted [67]. Similar results were also observed with a GFAP-tva model resulting in mainly mixed oligoastrocytomas. These mouse model studies collectively suggest that the cell of origin in gliomas depends on timing of transforming genetic alterations (embryonic or adult); the neuro-glial progenitor enriched regions in adults, but can also occur in differentiated astrocytes albeit with a lower efficiency.

## DISCUSSION

Mouse models of gliomagenesis have been pivotal in determining how the process of differentiation is integrally related to the process of gliomagenesis and the maintenance of the neoplastic phenotype. They indicate that not only the nature of transforming genetic alteration, but also the differentiation status of the neuro-glial cell, modulates gliomagenesis. Of interest, the oncogenic stimuli can themselves alter the state of neuro-glial differentiation, to make them more susceptible to transformation.

However, the question of ontogeny of gliomas remains in its infancy despite these mouse models. One of the problems is that the models to date target, using broadly expressed promoters such as Nestin and GFAP. For example, targeting the SVZ with CMV, GFAP or Nestin promoters target the stem and progenitor cells, but also differentiated glia that resides in this region. Use of cell specific promoters to drive expression of transforming alterations in neuro-glial cells at different stage of the normal lineage hierarchies is crucial, towards our understanding of initiating events in gliomagenesis within a defined cellular compartment. Identifying the cell of origin of gliomas and the region in the brain where these cells reside would permit a more systematic analysis of the genetic lesions involved in tumor initiation and progression. Moreover, the gene signature of the cell of origin may elucidate key molecular pathways and driver mutations that could lead to the development of new therapeutic approaches to prevent and target early stage disease. Finally, the integration of developmental biology, genomics and cancer biology will lead to the production of more accurate models, where single cell genetic manipulations in unambiguously labeled cells, will allow a better understanding of the ontogeny of gliomas.
